# Changes in Eating Habits and Sedentary Behavior During the COVID-19 Pandemic in Adolescents With Chronic Conditions

**DOI:** 10.3389/fped.2021.714120

**Published:** 2021-12-13

**Authors:** Bruna Caruso Mazzolani, Fabiana Infante Smaira, Camilla Astley, Amanda Yuri Iraha, Ana Jessica Pinto, Isabela Gouveia Marques, Milla Cordeiro Amarante, Nathalia Saffioti Rezende, Sofia Mendes Sieczkowska, Tathiane Christine Franco, Luana Cristina do Amaral Miranda, Lívia Lindoso, Alberto Carame Helito, Jane Oba, Ligia Bruni Queiroz, Rosa Maria R. Pereira, Hamilton Roschel, Clovis Artur Silva, Bruno Gualano

**Affiliations:** ^1^Applied Physiology and Nutrition Research Group, Rheumatology Division, School of Physical Education and Sport, Faculdade de Medicina (FMUSP), Universidade de São Paulo, São Paulo, Brazil; ^2^Laboratory of Assessment and Conditioning in Rheumatology, Disciplina de Reumatologia, Faculdade de Medicina (FMUSP), Universidade de São Paulo, São Paulo, Brazil; ^3^Instituto da Criança e do Adolescente, Hospital das Clinicas da Faculdade de Medicina (HCFMUSP), Universidade de São Paulo, São Paulo, Brazil; ^4^Rheumatology Division, Hospital das Clinicas da Faculdade de Medicina (HCFMUSP), Universidade de São Paulo, São Paulo, Brazil; ^5^Food Research Center, University of São Paulo, São Paulo, Brazil

**Keywords:** lifestyle, social distancing, pediatric, physical activity, youth

## Abstract

**Introduction:** Among healthy adolescents, school closures and home confinement were shown to increase unhealthier eating habits and sedentary behavior. It remains unknown to which extent the pandemic has impacted the lifestyle of adolescents with chronic conditions. Thus, the aim of this study is to report on the impact of the COVID-19 outbreak on eating habits and sedentary behavior among adolescents with multiple chronic conditions (*n* = 347) from a tertiary, referral hospital vs. healthy peers.

**Methods:** This observational study was conducted in São Paulo (Brazil) between July and October 2020, period in which a set of social distancing measures to contain the pandemic.

**Results:** The main findings of this study were that adolescents with chronic conditions and health peers showed important changes in eating habits (e.g., more often cooking and eating in front of television than before quarantine). Also, 86.8% of adolescents with chronic conditions and 91.6% of healthy adolescents reported increasing screen time during pandemic. No major differences were observed between patients and controls.

**Conclusions:** Adolescents with chronic conditions and healthy peers exposed to pandemic showed substantial changes in lifestyle, stressing the need for specific care to mitigate poor eating habits and excessive sedentary behavior for patients and healthy adolescents.

## Introduction

COVID-19 prevalence is around 2.9% for children and 2.2% for adolescents in Brazil (1). Although evidence shows that pediatric population is less susceptible to severe disease (2, 3), adolescents have been refrained from school, sports and social activities. Among healthy adolescents, school closures and home confinement were shown to increase unhealthier eating habits, such as increases in snacking, eating while watching television, and consumption of sweets and fried foods (4). Social distancing measures also scaled up sedentary behavior in teenagers (5, 6).

More strict social distancing measures have been specifically implemented among pediatric groups with chronic conditions (e.g., autoimmune diseases, gastrointestinal, kidney, and hepatic conditions), since they are deemed to be at risk for more severe COVID-19 (7, 8). Because of the pressure in the health system caused by the pandemic, some of these patients were also deprived of in-person medical assistance, resulting in sub-optimal health care delivery and limited recommendations regarding healthy lifestyle during the pandemic. However, it remains unknown to which extent the pandemic has impacted the lifestyle of adolescents with chronic conditions, among whom poorer eating habits and higher sedentariness could lead to even further overall health deterioration (9, 10).

This study reports on the impact of the COVID-19 pandemic on eating habits and sedentary behavior in a large cohort of adolescents with multiple chronic conditions vs. healthy peers.

## Methods

This is an observational study conducted between July and October 2020, during which a set of social distancing measures to contain the spread of COVID-19 were in place in São Paulo, Brazil. Participants were recruited from the Children's Institute of Clinical Hospital (School of Medicine of the University of São Paulo), the largest tertiary, referral, teaching hospital in Latin America. We invited 512 adolescents with chronic conditions (aged between 10 and 18 years) to participate in this study; 347 accepted the invitation and fulfilled the questionnaires. The group of patients comprised participants with juvenile rheumatic diseases [juvenile dermatomyositis (*n* = 23); juvenile idiopathic arthritis (*n* = 83); and childhood-onset systemic lupus erythematosus (*n* = 43)], gastrointestinal, and hepatic conditions [celiac disease (*n* = 12), eosinophilic esophagitis (*n* = 23), inflammatory bowel disease (*n* =44), autoimmune hepatitis (*n* = 28), and liver transplant (*n* = 50)], and kidney conditions [nephrotic syndrome (*n* = 22), chronic kidney disease (*n* = 7), and kidney transplant (*n* = 12)]. Additionally, 126 healthy adolescents, frequency-matched by age and sex, were recruited through social media and local newspapers to serve as controls; 95 met the eligibility criteria for controls (i.e., absence of pre-existing chronic conditions) and fulfilled the questionnaires. All participants completed an online survey on Research Electronic Data Capture^®^ (REDCap^®^) platform, which included questions about: (i) demographic characteristics (i.e., age, sex, ethnicity, and educational level) and changes in daily routine (e.g., school activities, home tasks, and leisure activities) during the pandemic; (ii) changes in eating habits during the pandemic (i.e., consumption of convenience foods and home-made meals, eating with others, eating in front of television/tablet/cellphone, mindful eating, and cooking), with possible answers being “more frequently,” “less frequently,” or “the same as before the pandemic”; (iii) time spent in sedentary behavior (i.e., any behavior characterized by a low energy expenditure while sitting or lying down when awaked) (11) during the pandemic assessed by screen time; (iv) changes in sedentary time during the pandemic.

Adolescents fulfilled the questionnaires in the presence of a parent and/or a research staff, and were instructed to ask for assistance if they had any difficulty in answering any question. Patients' disease status was assessed through medical records.

The study was approved by the Research Ethics Committee of Clinical Hospital (Approval Number: 31314220.5.0000.0068). The consent form was signed digitally by all adolescents and their legal guardians before the beginning of the survey.

### Statistical Analysis

Descriptive data are presented as mean and 95% confidence interval (95%CI) for continuous variables and absolute and relative frequency [*n* (%)] for categorical variables. Potential between-group differences for all dependent variables were tested by generalized estimating equations (GEE) models, based on the assumption of a multinomial distribution, cumlogit link function, and an exchangeable working correlation, with group as fixed factor. All GEE models were adjusted for age and sex. Analyses were performed using the statistical package SAS (version 9.4). The level of significance was set at *p* ≤ 0.05.

## Results

The mean age of patients and healthy adolescents was 14.3 years (95%CI: 14.0, 14.5) and 14.2 years (95%CI: 13.7, 14.7), respectively (*p* = 0.753). Baseline characteristics were comparable between groups (all *p* > 0.050). Most patients and controls were male (60.0 and 62.4%), Caucasians (60.0 and 51.4%) and attended public school (65.3 and 74.4%). Most adolescents reported changing daily routine after the implementation of social distancing measures (patients: 91.6%; controls: 92.8%; Table 1).

**Table 1:** General characteristics of participants.

	**Patients with chronic conditions (*n* = 347)**	**Healthy controls (*n* = 95)**	***p***
Age	14.3 (14.0–14.5)	14.2 (13.7–14.6)	0.753
**Sex**			
Male	217 (62.4)	57 (60.0)	0.675
Female	131 (37.6)	38 (40.0)	
**Self-reported ethnicity**			
Caucasian	179 (51.4)	57 (60.0)	0.139
Not-Caucasian	169 (48.6)	38 (40.0)	
**Current educational level**			
Not studying	9 (2.6)	4 (4.2)	0.784
Elementary school	207 (59.5)	56 (59.0)	
High school	114 (32.8)	29 (30.5)	
University	18 (5.2)	6 (6.3)	
**Type of school**			
Public	259 (74.4)	62 (65.3)	0.078
Private	89 (25.6)	33 (34.7)	
**Changed daily routine during the COVID-19 pandemic**			
Yes	323 (92.8)	87 (91.6)	0.684
No	25 (7.2)	8 (8.4)	

*Data presented as mean and 95% confidence interval (95%CI) or absolute and relative frequency [n (%)]*.

**Figure 1:**
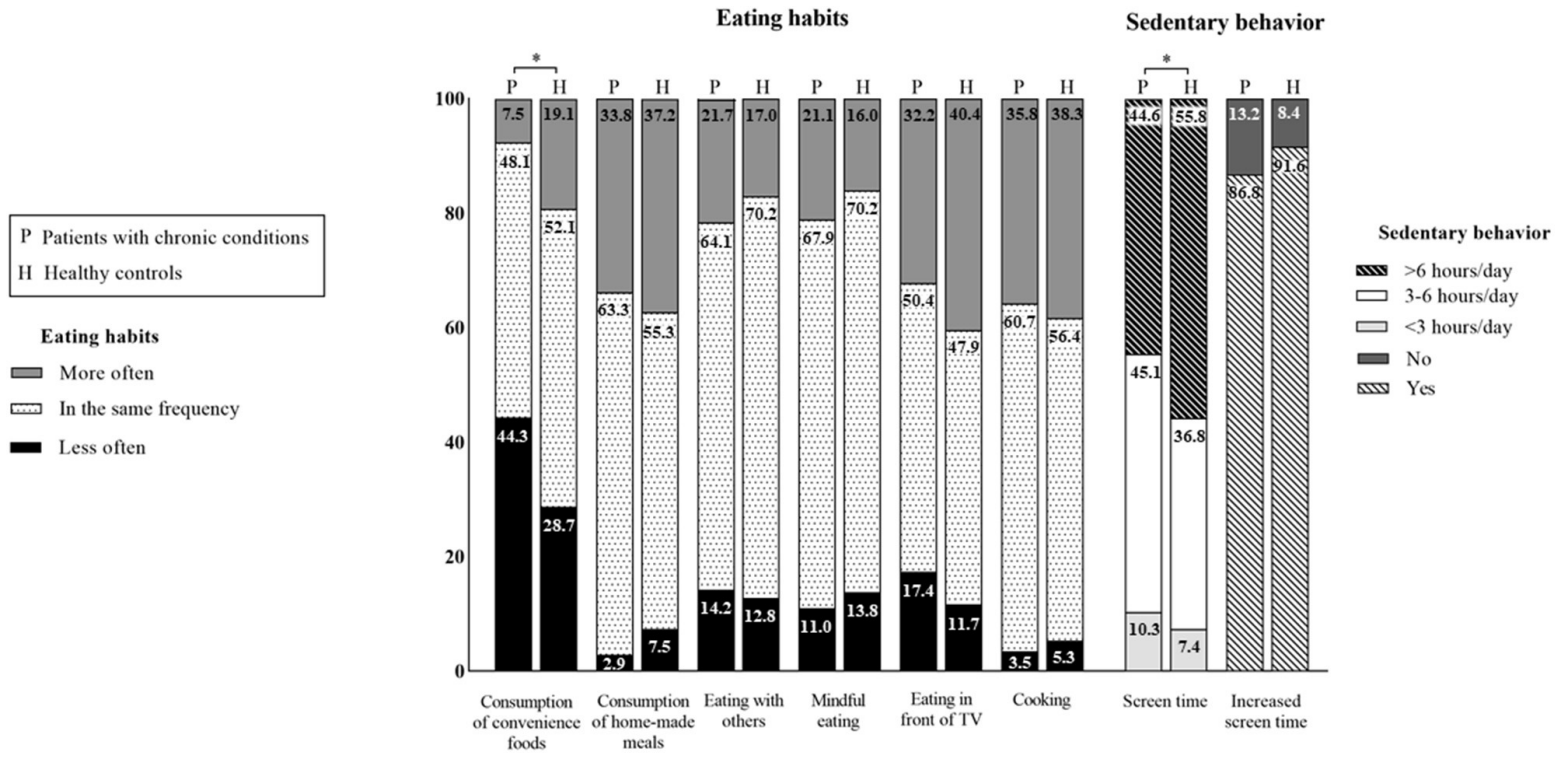
Changes in eating habits and sedentary behavior among adolescents with chronic conditions (*n* = 347) and healthy controls (*n* = 95) during the COVID-19 pandemic. *Significant difference between groups (*p* < 0.05).

Changes in eating habits in patients and controls during the COVID-19 pandemic are illustrated in Figure 1 (left panel). A small proportion of the adolescents with chronic conditions (7.5%) stated eating convenience foods more often than before quarantine, whereas 44.3% reported a less frequent consumption than before COVID-19 quarantine. The proportion of patients declaring consuming home-made meals more often than before quarantine was 33.8% vs. only 2.9% reporting a less often consumption than before quarantine. The percentage of patients reporting eating with others more often than before quarantine was 21.7%, while 14.2% declared doing so less often than before quarantine. While 21.1% of patients reported mindful eating more often than before quarantine, 11% stated doing it less frequently. An expressive percentage of the adolescents with chronic conditions (32.2%) reported eating in front of television more often than before quarantine vs. only 17.4% reporting doing so less frequently than before quarantine. More than a third (35.8%) of the patients reported participation in cooking more often than before quarantine vs. only 3.5% declaring doing so less frequently.

The proportions of patients and healthy controls did not differ for changes in the following eating habits during COVID-19: consumption of home-made meals (*p* = 0.933), eating with others (*p* = 0.612), eating in front of television/tablet/cellphone (*p* = 0.080), mindful eating (*p* = 0.198), and participation in cooking (*p* = 0.827) during the pandemic did not differ between groups. However, the proportion of patients and healthy controls changing consumption of convenience foods was significantly different (more often than before quarantine: 7.5 vs. 19.1%; less often than before quarantine: 44.3 vs. 28.7%, respectively, *p* < 0.001).

Regarding sedentary behavior (Figure 1, right panel), both adolescents with chronic conditions (86.8%) and their healthy counterparts (91.6%) reported increasing screen time during the pandemic (*p* = 0.219 for between-group comparison). Importantly, a greater part of the healthy adolescents' sample (55.8%) stated spending more than 6 h in sedentary behavior when compared with patients with chronic conditions (44.6%), whereas a greater proportion of patients declared spending 3–6 h (45.1%) and <3 h (10.3%) in sedentary behavior vs. healthy controls (36.8 and 7.4%, respectively) (*p* = 0.039).

## Discussion

To our knowledge, this is the first study to report on the impact of COVID-19 pandemic on lifestyle behaviors in a cohort of 347 adolescents with multiple chronic conditions. The main findings of this study were that adolescents with chronic conditions showed important changes in eating habits (e.g., less often consumption of convenience foods and more often eating in front of television than before quarantine) and substantial increase in screen time, a proxy of sedentary behavior, during the COVID-19 pandemic. Overall, changes in eating habits and sedentary behavior were similar across patients and controls.

The COVID-19 pandemic and the set of social distancing measures adopted to contain disease spread have been shown to impact eating habits worldwide (4, 12) which is also reflected in our sample. Some of these changes appear to be positive, such as the decrease in the frequency of convenience foods consumption and the increase in the proportion of consumption of home-made meals, eating with others, mindful eating and participation in cooking. Conversely, the increased proportion of eating in front of television is worrisome, as it has been associated with unhealth food choices (13–15) and poor healthy indexes (16). Considering that parents are generally responsible for providing food, making it available to eat and structuring when and where to eat (17), they should be informed on how to mitigate the potentially negative changes in eating habits brought about by COVID-19 pandemic.

The restrictive measures required to contain the COVID-19 pandemic affected all adolescents and raised a number of new challenges. Adolescence is a period of life characterized by the development of one's personality alongside several physiological and behavioral changes (18), with social interaction playing a major role. In this context, technology has emerged as a useful tool for adolescents to maintain socialization and school learning during the COVID-19 quarantine, helping attenuate the psychological adverse effects to this stressful period (18). However, the increased screen time came along with a dramatic raise in the proportion of adolescents (patients and controls alike) engaging in excessive sedentary behavior during the pandemic as observed herein, with approximately half of the participants reporting screen time over 6 h/day.

While these data align with others (5, 6) showing excessive sedentary behavior as a consequence of the pandemic, this is of particular relevance for clinical population. Adolescents are known to spend excessive time in sedentary behavior (19), which may be exacerbated in adolescents with chronic diseases (10, 20). In this specific group, hypoactivity has been considered a risk factor for poor clinical condition and worse symptoms (9, 11). Parents and healthcare providers should encourage physical activities and limit screen time whenever possible, particularly in the context of the pandemic. An interesting alternative is home-based exercise strategies. For instance, among adolescents with type 1 diabetes, this mode of training was shown to increase physical activity levels and help maintain or improve glycemic control (18, 21–23). As some chronic conditions (e.g., obesity, immunocompromised diseases) may aggravate COVID-19 prognosis (24, 25), forcing home confinement, home-based exercise programs may be a valuable and feasible alternative to maintain or increase physical activity levels (23, 26).

Strengths of this study involve the assessment of a relatively large cohort of patients with several chronic conditions during the most restrictive quarantine in Brazil. This study has limitations, however. The cross-sectional design does not allow inferring causative relationships between lifestyle changes and pandemic. Also, the use of self-reported questionnaire may reflect in some degree of imprecision on data reporting. Moreover, we were unable to measure physical activity, which could be a complementary information, although others have reported on this previously (27, 28). The relatively low number of patients for each disease did not allow for disease-specific sub-analysis. Finally, as a result of the suspension of face-to-face health care in our tertiary hospital (which was adapted to accommodate 1,000 hospital beds exclusively for COVID-19) (29), we were unable to conduct more in-depth, clinical and biochemical assessments.

In conclusion, adolescents with chronic conditions and healthy adolescents exposed to social distancing measures due to COVID-19 pandemic showed substantial changes in lifestyle. The findings from this study stress the need for specific care for healthy or diseased adolescents to mitigate poor eating habits and excessive sedentary behavior.

## Data Availability Statement

The raw data supporting the conclusions of this article will be made available by the authors, without undue reservation.

## Ethics Statement

The studies involving human participants were reviewed and approved by the Research Ethics Committee of Clinical Hospital (Approval Number: 31314220.5.0000.0068). Written informed consent to participate in this study was provided by the participants' legal guardian/next of kin.

## Author Contributions

BM and FS: responsible for study design, the collection, analysis, and interpretation of data, the writing of the report, and the decision to submit the manuscript for publication. CA, AI, IM, MC, NR, SS, TF, LM, LL, AH, JO, and LQ: responsible for study design and the collection of data, reviewing drafts of the article, and approval of the final version. AP: analysis and interpretation of data, the writing of the report, and approval of the final version. RP: responsible for study design, the writing of the report, and approval of the final version. HR, CS, and BG: analysis and interpretation of data, the writing of the report, and the decision to submit the manuscript for publication. All authors contributed to the article and approved the submitted version.

## Funding

This work was supported by São Paulo Research Foundation—FAPESP (Grants #2015/26937-4, #2019/14820-6, #2017/13552-2, #2015/03756-4, #2019/14819-8, #2019/20814-9, #2019/15231-4, #2016/00006-7); the Conselho Nacional de Desenvolvimento Científico e Tecnológico (CNPq 304984/2020-5; CNPQ 305556/2017-7); and the Núcleo de Apoio à Pesquisa Saúde da Criança e do Adolescente da USP (NAP-CriAd).

## Conflict of Interest

The authors declare that the research was conducted in the absence of any commercial or financial relationships that could be construed as a potential conflict of interest.

## Publisher's Note

All claims expressed in this article are solely those of the authors and do not necessarily represent those of their affiliated organizations, or those of the publisher, the editors and the reviewers. Any product that may be evaluated in this article, or claim that may be made by its manufacturer, is not guaranteed or endorsed by the publisher.
